# *Helicobacter pylori* Is Associated With Precancerous and Cancerous Lesions of the Gastric Cardia Mucosa: Results of a Large Population-Based Study in China

**DOI:** 10.3389/fonc.2020.00205

**Published:** 2020-03-03

**Authors:** Shuanghua Xie, Shaoming Wang, Liyan Xue, Daniel R. S. Middleton, Chentao Guan, Changqing Hao, Jinwu Wang, Bianyun Li, Ru Chen, Xinqing Li, Wenqiang Wei

**Affiliations:** ^1^National Cancer Registry Office, National Cancer Center/National Clinical Research Center for Cancer/Cancer Hospital, Chinese Academy of Medical Sciences and Peking Union Medical College, Beijing, China; ^2^Department of Pathology, National Cancer Center/National Clinical Research Center for Cancer/Cancer Hospital, Chinese Academy of Medical Sciences and Peking Union Medical College, Beijing, China; ^3^Section of Environment and Radiation, International Agency for Research on Cancer, Lyon, France; ^4^Department of Endoscopy, Cancer Institute/Hospital of Linzhou, Linzhou, China; ^5^Department of Pathology, Cancer Institute/Hospital of Linzhou, Linzhou, China; ^6^Department of Epidemiology, Cancer Institute/Hospital of Linzhou, Linzhou, China

**Keywords:** *Helicobacter pylori*, gastric cardia, precursor lesions, gastric cardia adenocarcinoma, ^13^C-urea breath test, population-based studies

## Abstract

**Background:** *Helicobacter pylori (H. pylori)* is widely accepted to be the most important cause of gastric non-cardia adenocarcinoma (GNCA), while its role in the development of gastric cardia adenocarcinoma (GCA) is not well-defined. We aimed to investigate current *H. pylori* infection in relation to the severity of both precancerous and cancerous lesions of the gastric cardia in an Asian population at high risk of GCA.

**Methods:** A population-based cross-sectional study was conducted in Linzhou County, Henan Province, China. Two thousand three (2,003) randomly selected participants with data on current *H. pylori* infection, assayed by ^13^C-urea breath test (^13^C-UBT), and a sequence of histological diagnoses of the gastric cardia mucosa were analyzed.

**Results:** Of 2,003 subjects, 828 (41.33%) were currently infected with *H. pylori*. The prevalence of current *H. pylori* infection increased with increasing severity of histological lesions, from 34.12% in subjects with normal gastric cardia mucosa to 52.17% in subjects with gastric cardia high-grade intraepithelial neoplasia (CHIN)/ gastric cardia adenocarcinoma (GCA) (*P* for trend <0.001). With *H. pylori*-negative subjects as the reference category, *H. pylori*-positive subjects had statistically significant elevated adjusted prevalence odds ratios (PORs) for each of the histological lesions. The PORs (95% CI) were 2.15 (1.74–2.64), 3.46 (2.08–5.75), 2.78 (1.90–4.07), and 3.05 (1.30–7.17) for subjects with carditis, cardia intestinal metaplasia (CIM), cardia low-grade intraepithelial neoplasia (CLIN), and CHIN/GCA), respectively. The associations remained when subjects with abnormal stomach non-cardia mucosa were excluded.

**Conclusions:** This large epidemiologic study demonstrates a positive association between current *H. pylori* infection and the severity of both precancerous and cancerous lesions of the gastric cardia in an Asian population at high risk of GCA. These findings suggest that *H. pylori* infection may play a role throughout both early- and late-stage development of GCA.

## Introduction

Gastric cancers are generally classified into two anatomical categories: gastric cardia adenocarcinoma (GCA), arising in the area of the stomach adjoining the esophageal–gastric junction, and gastric non-cardia adenocarcinoma (GNCA), arising from more distal regions of the stomach. Over the past few decades, while the incidence of GNCA has declined, the incidence of GCA has increased rapidly in Western countries (1–3), as well as in several Asian countries (4–7). According to recent global estimates (8), China accounts for nearly 52% of GCA cases worldwide with an estimated 135,000 new cases.

*Helicobacter pylori* (*H. pylori*), classified as a Group I carcinogen in 1994 by the International Agency for Research on Cancer (IARC) (9), is widely accepted to be the most important cause of GNCA. Various diagnostic methods are available to detect *H. pylori* infection and are usually divided into invasive (endoscopy-based) and non-invasive methods. Invasive diagnostic tests include endoscopic imaging, histology, rapid urease test, culture, and molecular methods. Non-invasive diagnostic tests include the urea breath test [^13^C-urea breath test (^13^C-UBT) or ^14^C-urea breath test], stool antigen test, and serological and molecular examinations (10). Of these, ^13^C-UBT is the preferred noninvasive approach to detect *H. pylori* in China (11), Japan (12), and other European societies (13).

Previously, *H. pylori* was believed to induce tissue responses in colonized hosts, a persistent process could further increase the risk of developing inflammation, intestinal metaplasia, dysplasia, and finally adenocarcinoma of the stomach non-cardia. However, the role of *H. pylori* infection in the development of precancerous and cancerous lesions of the gastric cardia is controversial. Epidemiologic studies focusing on the difference in seropositivity to *H. pylori* between GCA cases and controls have shown inconsistent results. Studies conducted in Western countries tend to show a null or even negative association (14–20), while in some high gastrointestinal cancer risk areas of the world, there is evidence of a higher risk of GCA among the infected (21–23).

Studies focused on precancerous lesions of the gastric cardia in relation to *H. pylori* infection may also improve our understanding of the biological mechanism of carcinogenesis and the natural history of GCA. Several studies have reported significant associations between *H. pylori* colonization—mainly assayed histologically or by rapid urease test—and inflammation (24–29) and intestinal metaplasia of the cardia (25–27, 29–31). Few studies have investigated the relationship with dysplasia of the cardia (28, 32), and no population-based study, to our knowledge, has simultaneously evaluated the association between *H. pylori* infection, assayed by ^13^C-UBT (a simple, accurate, non-invasive diagnostic test for current *H. pylori* infection) and the severity of both precancerous and cancerous lesions in the gastric cardia. We have had the opportunity of implementing such a study in an Asian population characterized by its high incidence of GCA, as well as low prevalence of reflux esophagitis and Barrett's metaplasia and early esophageal adenocarcinoma.

## Methods

### Study Design and Population

**Figure 1:**
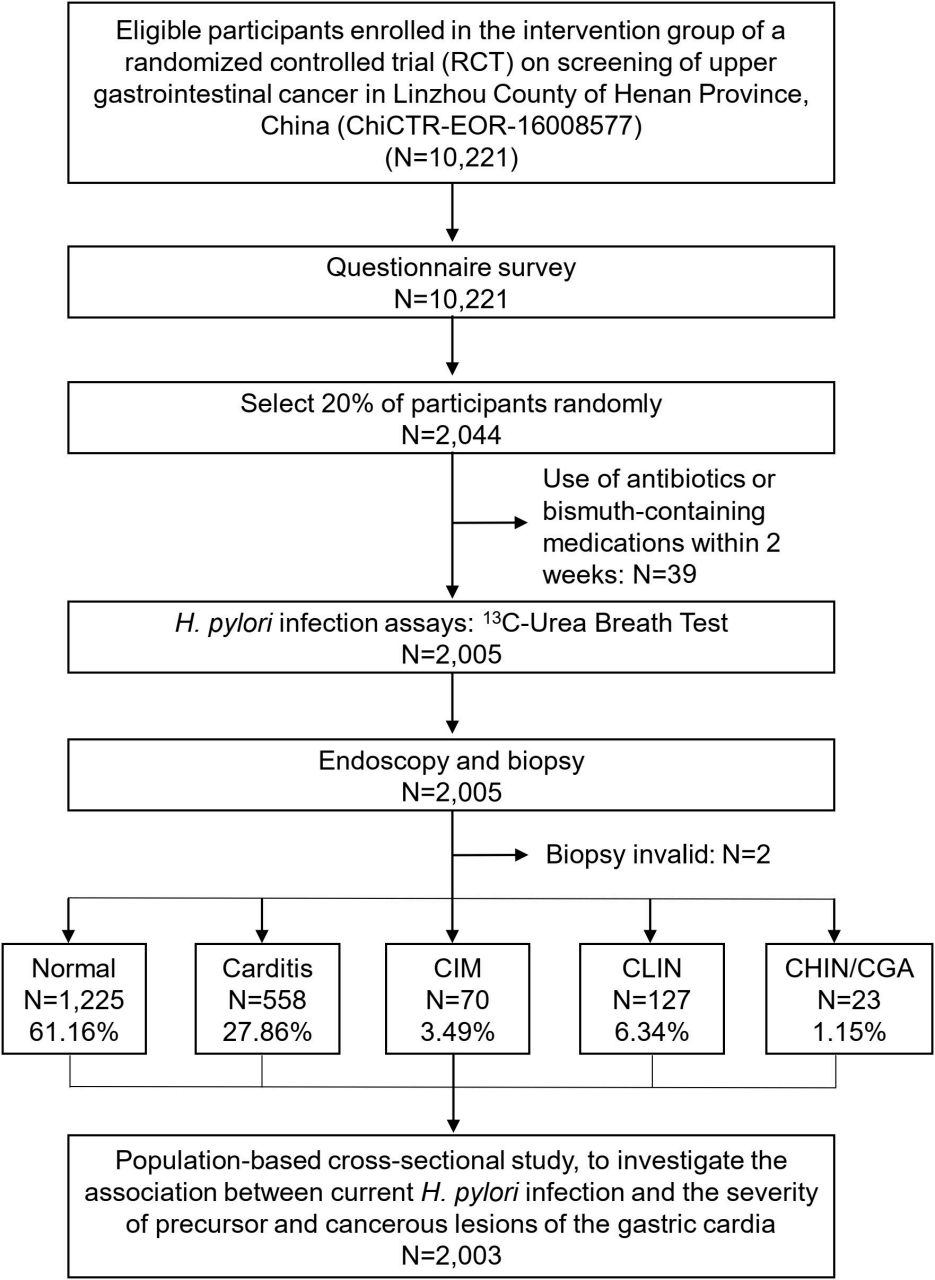
Flowchart of participants included in the study. Normal, normal mucosa; carditis, including superficial or chronic carditis with no intestinal metaplasia; CIM, cardia intestinal metaplasia; CLIN, cardia low-grade intraepithelial neoplasia; CHIN, cardia high-grade intraepithelial neoplasia; GCA, gastric cardia adenocarcinoma.

We conducted a randomized controlled trial (RCT) on screening of upper gastrointestinal cancers in Linzhou County, Henan Province, China, which has been previously reported elsewhere (33). This cross-sectional study was conducted only in a randomly selected population within the intervention arm of the RCT study (Figure 1). Briefly, villages regarded as clusters were randomly assigned by a computer-generated list to either an intervention group (screening by endoscopic examination) or a control group (with normal community care, i.e., only seeking screening when symptoms occurred) in a 1:1 ratio. From January 2014 to June 2016, 10,221 eligible men and women aged 40–69 years were enrolled in the intervention group. Of these, 2,044 (20%) were randomly selected by a computer-generated list for the ^13^C-UBT test. Inclusion criteria for the present study were: (1) local residents in selected villages; (2) aged 40–69 years; (3) no contraindications for endoscopic examination (e.g., history of reaction to iodine or lidocaine, serious cardiovascular disease, poor health status); (4) no history of cancer or endoscopic examination in the latest 3 years; and (5) providing informed consent to participate in screening. Exclusion criteria were: (1) participants with a history of liver cirrhosis, esophageal varices, hematemesis, a bleeding disorder, uncontrolled congestive heart failure, unstable angina, or a reaction to topical anesthetics or iodine; (2) participants having used antibiotics or bismuth-containing medications within 2 weeks prior to endoscopy. Of the initial 2,044 selected subjects, 39 subjects were excluded for not meeting the latter criterion. Thus, a total of 2,005 participants were included in the present study.

The study was approved by the ethics committee of the Cancer Institute and Hospital, Chinese Academy of Medical Sciences (approval number: 2015SQ00223), and the study used the Protocol Registration System in the Chinese Clinical Trial Registry (identifier: ChiCTR-EOR-16008577).

### Study Questionnaire

Before the ^13^C-UBT assay, each participant completed a standardized questionnaire administered by a trained interviewer. Information obtained included demographic characteristics, socioeconomic status, lifestyle habits (e.g., alcohol intake, cigarette smoking, dietary intakes, and drinking water source), medical history, and family medical history. After the interview, a basic physical examination was performed including the measurements of height and weight.

### ^13^C-Urea Breath Test

Eligible subjects underwent the ^13^C-UBT in the morning (between 8 a.m. and 10 a.m.) after an overnight fast (>8 h). A baseline breath sample (normal exhalation for 4 s) was collected in a collection tube. A capsule containing 50 mg ^13^C-urea in 50 ml of water was then administered orally. A second breath sample was collected 30 min later. Breath samples were then analyzed *via* mass spectrometry. Results were expressed as delta (Δ), defined as the ratio (r_i_−*r*_0_)/r_0_ where r = ^13^CO_2_/^12^CO_2_ (0 = basal sample; i = 30 min sample). The result of the ^13^C-UBT was considered positive for *H. pylori* infection if delta (Δ) was >4.0.

### Endoscopy

The entire esophagus and stomach were examined by esophagogastroduodenoscopy, including careful examinations of the cardia, especially focused on the high GCA incidence anatomical site located at the right anterior side of the esophagogastric junction (EGJ) from the axial view at endoscopy (34), according to a standardized protocol devised for the trial. The EGJ was recognized as the most proximal extent of the gastric fold at endoscopy. The cardia region was defined as the area 20 mm to the EGJ at endoscopy. During careful examinations of the stomach cardia and non-cardia (including the fundus, corpus, antrum, pylorus, and angle), suspicious lesions showing congestion, bleeding, roughness, erosion, plaque, or nodularity were targeted, and biopsies were taken. The number of biopsies taken was dependent on the size of the lesions (1~3 biopsies for each lesion). All endoscopic examinations were conducted in the local Cancer Institute/Hospital of Linzhou and were performed by board-certified endoscopists trained by the Cancer Institute/Cancer Hospital, Chinese Academy of Medical Sciences. Two endoscopists, blinded to the results of the questionnaire and ^13^C-UBT assay, performed the procedures. All endoscopic examinations were performed using the same type of endoscope (Olympus CV-260SL, Japan).

### Histopathology Examination

Biopsy specimens were fixed in 10% buffered formalin, embedded in paraffin, cut into 5-μm sections, and stained with hematoxylin and eosin (H&E) for histological examination according to a standard protocol. The presence of cardia mucosa was defined by the presence of mucous glands irrespective of the presence of oxyntic glands. One experienced gastrointestinal pathologist at the Cancer Institute/Hospital of Linzhou classified each cardia biopsy specimen as indicative of normal mucosa (Normal), carditis (including superficial or chronic carditis with no intestinal metaplasia), cardia intestinal metaplasia (CIM), cardia low-grade intraepithelial neoplasia (CLIN), cardia high-grade intraepithelial neoplasia (CHIN), and GCA (GCA including intramucosal cardia adenocarcinoma, submucosal cardia adenocarcinoma, and invasive cardia adenocarcinoma) according to a standardized protocol devised for the trial, which was drawn on the basis of the definitions in the World Health Organization (WHO) Classification of Tumors Pathology and Genetics of the Digestive System (2010) (35). The most severe disease classification indicated by any of the cardia biopsies was used as the global diagnosis for a given subject at each examination. Stomach non-cardia biopsy specimen was also classified as indicative of normal mucosa and abnormal mucosa (including superficial or chronic gastritis with no intestinal metaplasia, gastric intestinal metaplasia, gastric low-grade intraepithelial neoplasia, gastric high-grade intraepithelial neoplasia, and GNCA).

### Statistical Analyses

Body mass index (BMI) was calculated as body weight (in kilograms) divided by the square of height (in square meters). Education level was dichotomized as “middle school or above” vs. below. Smoking was defined as smoking at least one cigarette per day for more than 1 year. Alcohol consumption was defined as drinking at least one drink per day for more than 1 year. Pickled food intake and hot or rough food intake were defined as at least one consumption per week within the previous year. History of upper gastrointestinal disease included a history of reflux esophagitis, gastritis, and gastric or duodenal ulcer. Individuals with at least one relative diagnosed with cancer were defined as having a family history of cancer.

Categorical variables are presented as absolute and relative frequencies, and numerical variables are presented as means and standard deviations. Differences in categorical variables were examined using the chi-square test or Fisher's exact test, as appropriate. Cross-sectional analysis of the relationship between *H. pylori* and histological diagnosis was conducted by polytomous logistic regression. The Cochran–Armitage test was used for trends in the association between increasing severity of the histological lesions and current *H. pylori* infection rate. Covariates associated (*P* < 0.10) with *H. pylori* infection (sex, history of upper gastrointestinal disease) or histological lesions of the gastric cardia mucosa (age, sex, BMI, history of upper gastrointestinal disease) or deemed *a priori* as potential confounders (smoking, alcohol consumption, family history of cancer) were included in the multivariable polytomous logistic regression model. Thus, prevalence odds ratios (PORs) were adjusted for age (continuous), BMI (continuous), sex (male, female), smoking (yes, no), alcohol consumption (yes, no), history of upper gastrointestinal disease (yes, no), and family history of cancer (yes, no). A two-sided *P* value of <0.05 was considered statistically significant. For variables significantly associated with the study groups, pairwise comparisons to the reference group (Normal) were conducted using Pearson's chi-square test or the Fisher's exact test with statistical significance at *P* < 0.01. Statistical analyses were performed using SAS software, version 9.4 (SAS Institute Inc., Cary, NC, USA).

## Results

### Characteristics of the Study Population by Histological Lesions of Gastric Cardia Mucosa

Of 2,005 randomly selected subjects with both ^13^C-UBT and endoscopic examination, 983 (49%) with normal endoscopic findings were not targeted for biopsy and were classified as normal. The remaining 1,022 (51%) with suspicious endoscopic lesions were targeted, and biopsies were taken for histological examination. Two subjects were excluded from the present analysis because their biopsies did not provide a conclusive histological diagnosis. Thus, 2,003 participants were included in the present analysis, and the most advanced diagnosis was Normal for 1,225 (61.16%), carditis for 558 (27.86%), CIM for 70 (3.49%), CLIN for 127 (6.34%), and CHIN/GCA for 23 (1.15%) (Figure 1 and Table 1).

**Table 1:** Basic characteristics of the investigated population by histological lesions of the gastric cardia mucosa.

	**Normal**	**Carditis**	**CIM**	**CLIN**	**CHIN/GCA**
No.	1,225	558	70	127	23
Age (year), mean (SD)	53.30 (7.94)	54.61 (6.78)^a^	58.53 (6.87)^a^	57.55 (7.42)^a^	62.65 (5.27)^a^
BMI (kg/m^2^), mean (SD)	25.25 (3.57)	24.98 (4.12)	24.69 (3.01)	25.57 (3.96)	26.13 (5.72)
Sex, *n* (%) male	514 (41.96)	285 (51.08)^a^	34 (48.57)	69 (54.33)^a^	14 (60.87)
Education level ≥middle school, *n* (%)	815 (66.53)	368 (65.95)	43 (61.43)	74 (58.27)	11 (47.83)
Smoker ≥1 cigarette/day lasting ≥1 year, *n* (%)	261 (21.31)	141 (25.27)	16 (22.86)	37 (29.13)	7 (30.43)
Drinker ≥1 drink/day lasting ≥1 year, *n* (%)	66 (5.39)	32 (5.73)	1 (1.43)	4 (3.15)	0 (0.00)
Pickled food ≥1 time/week, *n* (%)	67 (5.47)	23 (4.12)	7 (10.00)	4 (3.15)	0 (0.00)
Hot or rough food ≥1 time/week, *n* (%)	313 (25.55)	161 (28.85)	17 (24.29)	24 (18.90)	3 (13.04)
History of upper gastrointestinal disease, yes, *n* (%)	164 (13.39)	85 (15.23)	4 (5.71)	13 (10.24)	4 (17.39)
Family history of cancer, yes, *n* (%)	590 (48.16)	259 (46.42)	35 (50.00)	57 (44.88)	9 (39.13)

Normal, normal mucosa; carditis, including superficial or chronic carditis with no intestinal metaplasia; CIM, cardia intestinal metaplasia; CLIN, cardia low-grade intraepithelial neoplasia; CHIN, cardia high-grade intraepithelial neoplasia; GCA, gastric cardia adenocarcinoma; BMI, body mass index.

a *P <0.01 with normal as the reference group*.

The mean ages of subjects with carditis, CIM, CLIN, and CHIN/GCA were significantly older than Normal (*P* < 0.01 for all). Subjects with carditis (51.08%) and CLIN (54.33%) were more likely to be males compared with those with normal mucosa (41.97%) (*P* < 0.01 for both). There were no significant differences in the severity of cardia histological lesions by BMI, education level, smoking, alcohol consumption, intake of pickled food, intake of hot or rough food, history of upper gastrointestinal disease, or family history of cancer (Table 1).

### Cross-Sectional Association of Precancerous and Cancerous Lesions and *Helicobacter pylori* Infection

Of 2,003 subjects, 828 (41.34%) had a positive *H. pylori* infection according to the ^13^C-UBT assay. None of the basic characteristics of the study population, including age, sex, education level, smoking, alcohol intake, intake of hot or rough food, intake of pickled food, family history of cancer, and BMI were statistically significantly associated with *H. pylori* exposure status (*P* > 0.05 for all), with the exception of history of upper gastrointestinal disease (*P* < 0.001) (Table 2).

**Table 2:** Current *Helicobacter pylori* infection rates assayed by ^13^C-urea breath test by basic characteristics of the investigated population in Linzhou, China.

	***H. pylori* (–)** ***n* (%)**	***H. pylori* (+)** ***n* (%)**	***P^**a**^***
Age (year)			0.380
≥55	537 (59.73)	362 (40.27)	
<55	638 (57.79)	466 (42.21)	
Sex			0.073
Female	618 (56.85)	469 (43.15)	
Male	557 (60.81)	359 (39.19)	
Education level			0.213
≥Middle school	756 (57.67)	555 (42.33)	
< Middle school	419 (60.55)	273 (39.45)	
Smoker ≥1 cigarette/day lasting ≥1 year			0.913
Yes	270 (58.44)	192 (41.56)	
No	905 (58.73)	636 (41.27)	
Drinker ≥1 drink/day lasting ≥1 year			0.906
Yes	61 (59.22)	42 (40.78)	
No	1,114 (58.63)	786 (41.37)	
Pickled food ≥1 time/week			0.796
Yes	58 (57.43)	43 (42.57)	
No	1,117 (58.73)	785 (41.27)	
Hot or rough food ≥1 time/week			0.825
Yes	306 (59.07)	212 (40.93)	
No	869 (58.52)	616 (41.48)	
History of upper gastrointestinal disease			<0.001
Yes	191 (70.74)	79 (29.26)	
No	984 (56.78)	749 (43.22)	
Family history of cancer			0.631
Yes	552 (58.11)	398 (41.89)	
No	623 (59.16)	430 (40.84)	
BMI (kg/m^2^)			0.422
<25.0^b^	528 (57.27)	394 (42.73)	
≥25.0 and <30.0	534 (60.27)	352 (39.73)	
≥30.0	113 (57.95)	82 (42.05)	

BMI, body mass index.

a Chi-square test.

b *Including 20 subjects with BMI <18.5 kg/m^2^*.

*Helicobacter pylori* positivity rates were 50.90% for subjects with carditis (*P* < 0.001), 61.43% for subjects with CIM (*P* < 0.001), 55.91% for subjects with CLIN (*P* < 0.001), and 52.17% for subjects with CHIN/GCA (*P* = 0.071) vs. 34.12% for subjects with normal mucosa. With increasing severity of histological lesions in the cardia, *H. pylori* positivity rate increased significantly (*P* for trend <0.001) (Table 3). After excluding individuals with abnormal stomach non-cardia mucosa (*N* = 452), *H. pylori* positivity rates were 30.58%, 48.11%, 61.02%, 56.00%, and 50.00% for Normal, carditis, CIM, CLIN, and CHIN/GCA, respectively, and also showed a trend with increasing severity of histologic lesions (*P* for trend <0.001) (Supplementary Table 1).

**Table 3:** Current *Helicobacter pylori* infection rates, assayed by ^13^C-urea breath test, by the severity of precancerous and cancerous lesions of gastric cardia mucosa of the investigated population in Linzhou, China.

**Histological** **Lesions**	***H. pylori* (–)** ***n* (%)**	***H. pylori* (+)** ***n* (%)**	***P^**a**^***
Normal	807 (65.88)	418 (34.12)	Reference
Carditis	274 (49.10)	284 (50.90)	<0.001
CIM	27 (38.57)	43 (61.43)	<0.001
CLIN	56 (44.09)	71 (55.91)	<0.001
CHIN/GCA	11 (47.83)	12 (52.17)	0.071
			<0.001^b^

Normal, normal mucosa; carditis, including superficial or chronic carditis with no intestinal metaplasia; CIM, cardia intestinal metaplasia; CLIN, cardia low-grade intraepithelial neoplasia; CHIN, cardia high-grade intraepithelial neoplasia; GCA, gastric cardia adenocarcinoma; H. pylori, Helicobacter pylori.

a Chi-square test with subjects with normal as the reference group.

b *Cochran–Armitage trend tests*.

**Figure 2:**
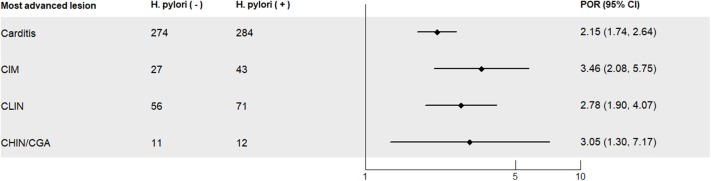
Prevalence odds ratios (PORs) for severity of precancerous and cancerous lesions of the gastric cardia in the investigated study population in Linzhou, China, with *Helicobacter pylori*-negative subjects as the reference. Normal, normal mucosa; carditis, including superficial or chronic carditis with no intestinal metaplasia; CIM, cardia intestinal metaplasia; CLIN, cardia low-grade intraepithelial neoplasia; CHIN, cardia high-grade intraepithelial neoplasia; GCA, gastric cardia adenocarcinoma. PORs, prevalence odds ratios; CI, confidence interval; PORs were calculated for each level of lesions, with *H. pylori*-negative subjects as the control group, and PORs were adjusted for age (continuous), BMI (continuous), sex (male, female), smoking (yes, no), alcohol consumption (yes, no), history of upper gastrointestinal disease (yes, no), and family history of cancer (yes, no).

With *H. pylori* negative subjects as the reference, *H. pylori*-positive subjects had statistically significant elevated PORs for each of the histological lesions after adjusting for sex, age, smoking, alcohol intake, BMI, history of upper gastrointestinal disease, and family history of cancer [for carditis: POR = 2.15 (95% CI: 1.74–2.64); for CIM: POR = 3.46 (95% CI: 2.08–5.75); for CLIN: POR = 2.78 (95% CI: 1.90–4.07); for CHIN/GCA: POR = 3.05 (95% CI: 1.30–7.17)] (Figure 2). The PORs increased when subjects with abnormal stomach non-cardia mucosa were excluded (Supplementary Figure 1).

## Discussion

To our knowledge, this is the first study to show a significant positive association between current *H. pylori* infection assayed by ^13^C-UBT and the severity of both precancerous and cancerous lesions in gastric cardia in an Asian population at high risk of GCA. The prevalence of current *H. pylori* infection increased with increasing severity of histological lesions in the gastric cardia, from 34.12% in subjects with normal gastric cardia mucosa to 61.43% in subjects with CIM, 55.91% in subjects with CLIN, as well as 52.17% in subjects with CHIN or advanced lesions. With *H. pylori*-negative subjects as the reference category, *H. pylori*-positive subjects had statistically significant elevated adjusted PORs for each of the histological lesions. In addition, the associations held when the analysis was conducted among subjects with normal stomach non-cardia.

Since its initial isolation in 1983, *H. pylori* has generally been believed to play an important role in the process of GNCA carcinogenesis, with a large body of evidence showing a strong positive association between *H. pylori* infection and GNCA. However, the role of *H. pylori* in the development of GCA is less well-clarified with a controversial and relatively small body of evidence.

A number of previous epidemiological studies conducted in Asian populations, such as China (21, 36–39), Korea (23, 40, 41), Japan (42–44), and Iran (45) found that seropositivity to *H. pylori* was associated with a significantly increased risk of GCA. A recent systematic review of studies conducted among Koreans reported that *H. pylori* infection was associated with a 2.88-fold higher risk of GCA (46), and another meta-analysis reported a positive association between *H. pylori* infection and GCA in geographic regions with a high incidence of GCA (22). The present study explored, for the first time, the association between current *H. pylori*, assayed by ^13^C-UBT, and the severity of precancerous and cancerous lesions in the gastric cardia. The finding of a positive association with CHIN/GCA is consistent with earlier reports in Linzhou (21, 38, 39), as well as in the other abovementioned Asian studies (23, 42, 43, 45, 47). However, this is contradictory to Western studies (14, 15, 17, 19), where the association with GCA tended to be null or inverse. One explanation for the geographic variation in the association between *H. pylori* and GCA could be the differences among study populations in the definition of cardia cancer and disease etiology, particularly with respect to the prevalence of gastroesophageal reflux disease (GERD) (48). In most Western populations, Barrett's esophagus and adenocarcinoma of the esophagus are relatively common (49). However, in the present study conducted in Linzhou, as well as other regions of Asia, Barrett's esophagus and adenocarcinoma of the esophagus are very rare (49). Adenocarcinomas classified as cardia adenocarcinomas do not or rarely include esophageal adenocarcinoma cases, which appear to be positively associated with GERD and inversely associated with *H. pylori* infection (48, 50). Alternatively, differences in the genetics of *H. pylori* or host genetics in populations from different geographic regions may also explain part of these discrepancies (45, 51). Although the present study did not include advanced GCA (most GCA cases in CHIN/GCA category were early GCA including intramucosal cardia adenocarcinoma, submucosal cardia adenocarcinoma through endoscopic screening), it revealed that the prevalence of current *H. pylori* infection in subjects with advanced precancerous and early cancerous lesions in the gastric cardia (52.17%) was higher vs. that in normal controls (34.12%). Furthermore, there was an increasing trend of current *H. pylori* infection prevalence rate with increasing severity of histologic lesions in gastric cardia (*P* for trend <0.001). Current *H. pylori* positivity rate was positively associated with the severity of both precancerous and cancerous lesions in the gastric cardia after adjusting for possible confounding factors.

A number of gastroscopic surveys have also found an association between *H. pylori* infection, assayed histologically or with the urease test, and carditis or CIM (24–26, 31, 52, 53). In comparison to these previous studies, the present study is unique in assaying current *H. pylori* infection status using ^13^C-UBT—a simple, accurate, and non-invasive diagnostic test for current *H. pylori* infection in a relatively large number of subjects (*n* = 2,003) with a broad range of precancerous and cancerous lesions in the gastric cardia. Furthermore, the associations remained when subjects with abnormal stomach non-cardia mucosa were excluded from the analysis. The microbiological basis for the observed positive associations between current *H. pylori* infection and histological lesions of the gastric cardia is not entirely clear. However, one speculative explanation is that *H. pylori* induces persistent tissue responses in colonized cardia mucosa, and the persistent process may increase the risk of developing inflammation, atrophy, intestinal metaplasia, intraepithelial neoplasia, and adenocarcinoma of the gastric cardia (39, 48, 54). As this study is limited by its cross-sectional design, further prospective epidemiology studies and experiment research will be necessary to confirm the associations and elucidate their underlying biological mechanism.

Our results are worth consideration for several reasons. Firstly, to our knowledge, our study is the first to simultaneously evaluate the association between current *H. pylori* infection, assayed by ^13^C-UBT, and a broad range of histological lesions in the gastric cardia in an Asian population with a high incidence of GCA. Secondly, the relatively large sample size recruited from a general population in an area with a high incidence of GCA and rarity of GERD, Barrett's metaplasia, or early esophageal adenocarcinoma enabled us to explore current *H. pylori* infection in relation to a broad range of histologic lesions of the cardia. Targeted endoscopic examination of the high-risk site in the cardia and biopsies taken from the cardia and any other suspicious lesions in the stomach non-cardia were also strengths of the study design.

We acknowledge some limitations of the study design. We have no data on previous *H. pylori* infection and prior attempts at *H. pylori* eradication in study participants. However, we do not believe that eradication therapy could have substantially changed our estimates, as eradication therapy is not a public health campaign in Linzhou. Furthermore, although ^13^C-UBT is the recommended noninvasive approach to detect *H. pylori* with high sensitivity and specificity, it could not provide us strain-specific *H. pylori* data (e.g., CagA and VacA) as prior studies have reported that CagA- and VacA-positive *H. pylori* are more virulent than negative strains (45). The study was also limited by its cross-sectional design, which does not allow reverse causality to be ruled out. Future prospective evidence from our follow-up results and other studies will be valuable in gaining further insight on the associations between *H. pylori* infection and histological lesions of the gastric cardia.

In conclusion, our findings indicated that current infection with *H. pylori* was positively associated with the severity of both precancerous and cancerous lesions of the gastric cardia in an Asian population at high risk of GCA. Further research from other locations and populations is needed to reveal the role of *H. pylori* infection in the development of GCA.

## Data Availability Statement

The datasets for this article are not publicly available because all our data are under regulation of both the National Cancer Center of China and Cancer Institute/Hospital of Linzhou. Requests to access the datasets should be directed to Wenqiang Wei (weiwq@cicams.ac.cn) and Changqing Hao (haochq@126.com).

## Ethics Statement

The studies involving human participants were reviewed and approved by the Ethics Committee of Cancer Institute and Hospital, Chinese Academy of Medical Sciences (Approval No: 2015SQ00223). The patients/participants provided their written informed consent to participate in this study.

## Author Contributions

WW conceived the study and acquired funding. CH, LX, JW, and BL conducted fieldwork in Linzhou, including endoscopic screening, histological diagnosis, epidemiological surveys, *H. pylori* infection assays, and data acquisition. XL was responsible for the quality control of endoscopy, histological analysis, and data collection. SX, CG, and RC oversaw data collection and management. SX, SW, and DM performed statistical analysis. SX led the authorship of the manuscript. All authors contributed to the preparation, writing, and review of the manuscript.

### Conflict of Interest

The authors declare that the research was conducted in the absence of any commercial or financial relationships that could be construed as a potential conflict of interest.

## Supplementary Material

Supplementary Figure 1 Prevalence odds ratios (PORs) for severity of precancerous and cancerous lesions of the gastric cardia among subjects with normal non-cardia stomach in Linzhou, China, with *H. pylori* negative subjects as the reference. Normal, normal mucosa; Carditis, including superficial or chronic carditis with no intestinal metaplasia; CIM, cardia intestinal metaplasia; CLIN, cardia low-grade intraepithelial neoplasia; CHIN, cardia high-grade intraepithelial neoplasia; GCA, gastric cardia adenocarcinoma; PORs, Prevalence odds ratios; Cl, confidence interval. PORs were calculated for each level of lesions, with *H. pylori* negative subjects as the control group, and PORs were adjusted for age (continuous), BMI (continuous), sex (male, female), smoking (yes, no), alcohol consumption (yes, no), history of upper gastrointestinal disease (yes, no), and family history of cancer (yes, no).

Supplementary Table 1 Current *Helicobacter pylori* infection rates, assayed by 13C-UBT, by the severity of precancerous and cancerous lesions of gastric cardia mucosa among subjects with normal non-cardia stomach in Linzhou, China. Normal, normal mucosa; Carditis, including superficial or chronic carditis with no intestinal metaplasia; CIM, cardia intestinal metaplasia; CLIN, cardia low-grade intraepithelial neoplasia; CHIN, cardia high-grade intraepithelial neoplasia; CGA, cardia gastric adenocarcinoma; *H. pylori, Helicobacter pylori*. ^1^Chi-Square test with subjects with normal as the reference group; ^2^Cochran-Armitage trend tests.

## Abbreviations

BMI: body mass index
GCA: gastric cardia adenocarcinoma
CHIN: cardia high-grade intraepithelial neoplasia
CIM: cardia intestinal metaplasia
CLIN: cardia low-grade intraepithelial neoplasia
^13^C-UBT: ^13^C-urea breath test
ESCC: esophageal squamous cell carcinoma
*H. pylori*: *Helicobacter pylori*
GNCA: gastric non-cardia adenocarcinoma
POR: prevalence odds ratio
RCT: randomized controlled trial.
