# Supplementary figures and images for: Helicobacter pylori Is Associated With Precancerous and Cancerous Lesions of the Gastric Cardia Mucosa: Results of a Large Population-Based Study in China
Source: Front Oncol. 2020 Mar 3;10:205. doi: 10.3389/fonc.2020.00205 (PMC7063085; doi:10.3389/fonc.2020.00205)

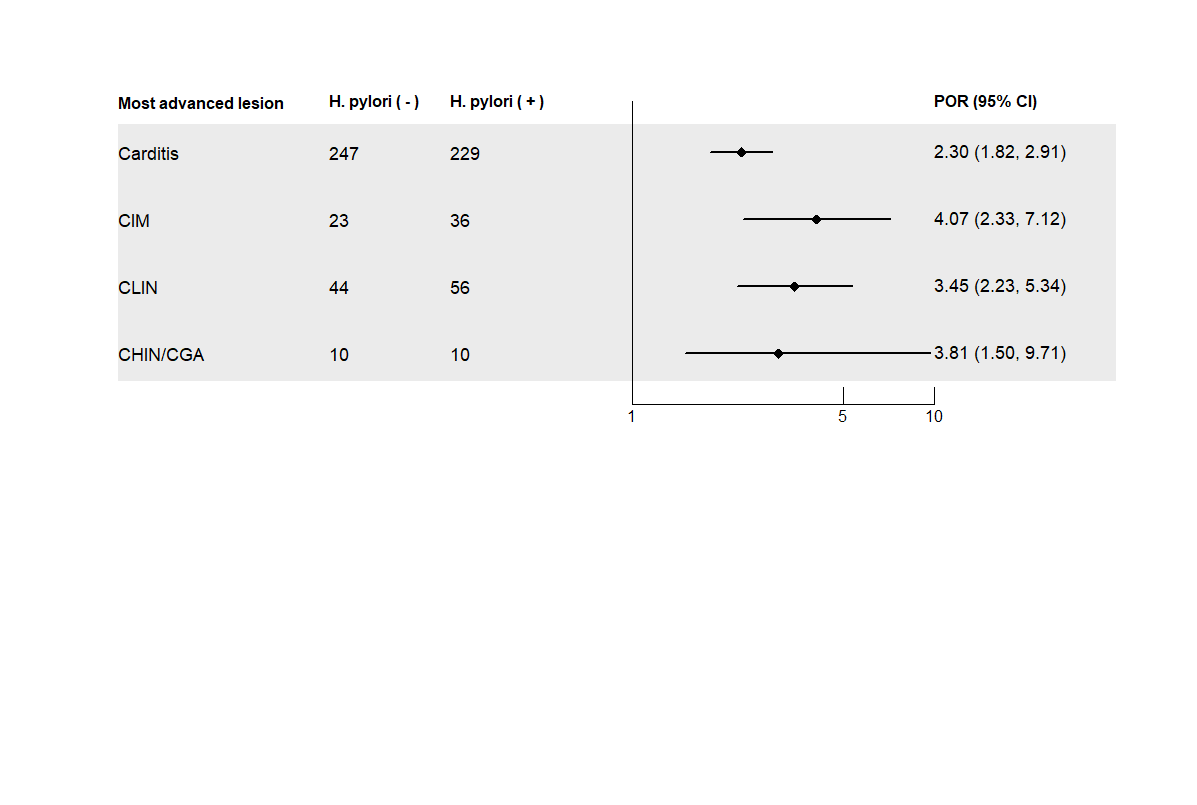

Supplement: Supplementary Figure 1 — Prevalence odds ratios (PORs) for severity of precancerous and cancerous lesions of the gastric cardia among subjects with normal non-cardia stomach in Linzhou, China, with H. pylori negative subjects as the reference. Normal, normal mucosa; Carditis, including superficial or chronic carditis with no intestinal metaplasia; CIM, cardia intestinal metaplasia; CLIN, cardia low-grade intraepithelial neoplasia; CHIN, cardia high-grade intraepithelial neoplasia; GCA, gastric cardia adenocarcinoma; PORs, Prevalence odds ratios; Cl, confidence interval. PORs were calculated for each level of lesions, with H. pylori negative subjects as the control group, and PORs were adjusted for age (continuous), BMI (continuous), sex (male, female), smoking (yes, no), alcohol consumption (yes, no), history of upper gastrointestinal disease (yes, no), and family history of cancer (yes, no). [file Image_1.tiff]
