# Supplementary material for: Helicobacter pylori Is Associated With Precancerous and Cancerous Lesions of the Gastric Cardia Mucosa: Results of a Large Population-Based Study in China
Source: Front Oncol. 2020 Mar 3;10:205. doi: 10.3389/fonc.2020.00205 (PMC7063085; doi:10.3389/fonc.2020.00205)
Supplement: Supplementary Table 1 — Current Helicobacter pylori infection rates, assayed by 13C-UBT, by the severity of precancerous and cancerous lesions of gastric cardia mucosa among subjects with normal non-cardia stomach in Linzhou, China. Normal, normal mucosa; Carditis, including superficial or chronic carditis with no intestinal metaplasia; CIM, cardia intestinal metaplasia; CLIN, cardia low-grade intraepithelial neoplasia; CHIN, cardia high-grade intraepithelial neoplasia; CGA, cardia gastric adenocarcinoma; H. pylori, Helicobacter pylori. 1Chi-Square test with subjects with normal as the reference group; 2Cochran-Armitage trend tests. [file Table_1.doc]

| **Supplementary Table 1.** Current *Helicobacter pylori* infection rates, assayed by ^13^C-UBT, by the severity of precancerous and cancerous lesions of gastric cardia mucosa among subjects with normal non-cardia stomach in Linzhou, China | | | |
| --- | --- | --- | --- |
| **Histological**  **lesions** | ***H. pylori* ( - )**  **n (%)** | ***H. pylori* ( + )**  **n (%)** | ***P^1^*** |
| Normal | 622 (69.42) | 274 (30.58) | ref |
| Carditis | 247 (51.89) | 229 (48.11) | <0.001 |
| CIM | 23 (38.98) | 36 (61.02) | <0.001 |
| CLIN | 44 (44.00) | 56 (56.00) | <0.001 |
| CHIN/CGA | 10 (50.00) | 10 (50.00) | 0.063 |
|  |  |  | <0.001^2^ |
| Normal, normal mucosa; Carditis, including superficial or chronic carditis with no intestinal metaplasia; CIM, cardia intestinal metaplasia; CLIN, cardia low-grade intraepithelial neoplasia; CHIN, cardia high-grade intraepithelial neoplasia; CGA, cardia gastric adenocarcinoma. H. pylori, Helicobacter pylori. ^1^ Chi-Square test with subjects with normal as the reference group; ^2^ Cochran-Armitage trend tests. | | | |
